# Three-Dimensional Texture Analysis Based on PET/CT Images to Distinguish Hepatocellular Carcinoma and Hepatic Lymphoma

**DOI:** 10.3389/fonc.2019.00844

**Published:** 2019-09-03

**Authors:** Hanyue Xu, Wen Guo, Xiwei Cui, Hongyu Zhuo, Yinan Xiao, Xuejin Ou, Yunuo Zhao, Tao Zhang, Xuelei Ma

**Affiliations:** ^1^State Key Laboratory of Biotherapy and Cancer Center, West China Hospital, Sichuan University and Collaborative Innovation Center for Biotherapy, Chengdu, China; ^2^West China School of Medicine, West China Hospital, Sichuan University, Chengdu, China; ^3^West China Hospital, Sichuan University, Chengdu, China

**Keywords:** hepatocellular carcinoma, hepatic lymphoma, positron emission tomography–computed tomography, texture, differentiation

## Abstract

**Objectives:** This study compared the diagnostic ability of image-based parameters with texture parameters in the differentiation of hepatocellular carcinoma (HCC) and hepatic lymphoma (HL) by positron emission tomography–computed tomography (PET/CT).

**Methods:** Patients with pathological diagnosis of HCC and HL were included in this study. Image-based and texture parameters were obtained by manual drawing of region of interest. Receiver operating characteristic (ROC) was used to test the diagnostic capacity of each parameter. Binary logistic regression was used to transform the most discriminative image-based parameters, texture parameters, and the combination of these parameters into three regression models. ROC was used to test the diagnostic capacity of these models.

**Result:** Ninety-nine patients diagnosed with HCC (*n* = 76) and HL (*n* = 23, 10 primary HL, 13 secondary HL) by histological examination were included in this study (From 2011 to 2018, West China hospital). According to the AUC and *p*-value, 2 image-based parameters and five texture parameters were selected. The diagnostic ability of texture-based model was better than that of image-based model, and after combination of those two groups of parameters the diagnostic capacity improved.

**Conclusion:** Texture parameters can differentiate HCC from HL quantitatively and improve the diagnostic ability of image-based parameters.

## Introduction

Malignant hepatic nodules include primary malignant hepatic neoplasms, such as hepatocellular carcinoma (HCC) and intrahepatic cholangiocarcinoma (ICC), and metastatic diseases from bile duct, lymphoid cells, endothelial cells. Hepatocellular carcinoma (HCC) accounts for almost 80% of all primary malignant hepatic neoplasms (1). Primary hepatic lymphoma (PHL) is rare, while secondary hepatic lymphoma (SHL), widespread lymphoma with liver involved, appears in 50% patients with non-Hodgkin lymphoma and 20% patients with Hodgkin lymphoma (2).

Differentiation between HCC and hepatic lymphoma, both in its primary and secondary form, is difficult. The images of HL is variable and can be similar to that of HCC, and several studies have reported cases about patients with PHL mimicking HCC (3–5). SHL is characterized by hepatosplenomegaly and systemic involvement which contributes to clinic diagnosis, but radiological diagnosis of SHL still requires further improvement since it has no specific features in sonography, and can appear as a solitary lesion (2, 6). Although the Biopsy has been extensively used as a clinic tool to distinguish HCC from HL, cancer cells extracted only represent part of the lesion and therefore may cause selection bias. Positron emission tomography-computed tomography (PET/CT), a non-invasive clinic examination, reveals the anatomical structure as well as the glucose metabolism of tissues and therefore has been widely used in the diagnosis and prognosis of hepatic lesions (7, 8). However, the accuracy of PET/CT in the diagnosis of different hepatic lesions remains to be improved (9).

Texture analysis is a newly-developed high throughput way to extract digital information from images that naked eyes cannot detect, and can thus explore more characteristics of images (10). Some studies have adopted texture analysis combined with PET/CT to differentiate benign from malignant mediastinal lymph nodes and distinguish malignant from benign bone and soft-tissue lesions (11, 12). Aiming at distinguishing HCC from HL in a non-invasive way, we explored the ability of texture and image-based parameters of PET/CT in differentiating HCC and HL.

## Methods

### Patients

This study was approved by the West China Hospital Ethics Committee and had a waiver of patients written informed consent. From Jan. 2011 to Dec. 2018, all patients diagnosed with liver lesions by PET/CT were recruited, and only patients with pathological diagnosis of HCC and HL were included. Their information was obtained from the clinical and radiological databases of our hospital. The inclusion criteria were having: (1) images obtained before treatment for hepatic lesions, (2) images obtained from a same system, (3) complete clinical and radiological information, (4) pathological diagnosis of HCC or HL; exclusion criteria were having: (1) incomplete image or clinical information, (2) FDG uptake of liver lesions below or comparable to background activity, (3) liver transplantation.

### PET/CT Examination

Patients were fasted 4–6 h and had serum glucose concentration <200 mg/dl before the intravenous injection of 185–370 MBq of 18F-FDG (4 MBq/kg of body weight). After injection, patients rested in a quiet room for 1 h. Then, a whole-body PET/CT scanner (Gemini GXL; Philips Medical Systems, The Netherlands) was used for imaging. During imaging process, patients were in supine position with both arms extended in the cranial direction and breathing quietly. PET images were obtained at 2 min/bed.

The CT images were acquired simultaneously with parameters as follows: 40 mAs, 120 kVp, a slice thickness of 2 mm, and a pitch of 4 mm. After acquisition completed, the transverse, sagittal, and coronal plane images of CT and PET were reconstructed automatically by the computer. The PET images were reconstructed by the line of response (LOR) method after a CT attenuation correction.

### Radiomics Extraction

**Figure 1:**
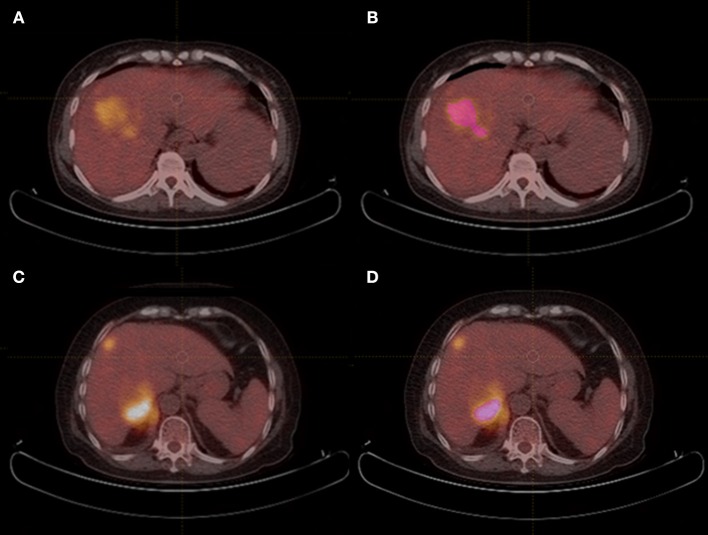
PET/CT images of HCC and HL case examples. **(A,B)** An example of hepatocellular carcinoma mimicking hepatic lymphoma, and region of interest was drawn in **(B)**. (CONVENTIONAL_SUVmin = 2.1, CONVENTIONAL_TLG = 293.5, SHAPE_Compacity = 2.41, GLCM_Correlation = 0.637, GLRLM_GLNU = 105.7, NGLDM_Contrast=0.055, and GLZLM_GLNU=14.4); **(C,D)** An example of secondary hepatic lymphoma mimicking hepatocellular carcinoma, and region of interest was drawn in **(D)**. (CONVENTIONAL_SUVmin = 6.6, CONVENTIONAL_TLG = 425.9, SHAPE_Compacity = 1.98, GLCM_Correlation = 0.567, GLRLM_GLNU = 43.4, NGLDM_Contrast=0.238, and GLZLM_GLNU = 18.9).

All scans were analyzed by two senior residents independently (HYX, 3 year of training; WG, 4 year of training) and were supervised by a senior radiologist (XLM, 13 years of experience) in order to handle the non-consensus. All of them were blinded to the histological outcomes. Each region of interest (ROI) was manually drawn along the liver lesion, slice by slice on axial images, by using a dedicated software for image analysis (LIFEx software, version 3.74, French Alternative Energies and Atomic Energy Commission). Figure 1 shows PET/CT images of two case examples, HCC and HL, respectively. Intra-luminal water, cavity, and necrotic components that can be distinguished from tumor solid portion by naked eye were excluded via a fixed 40% threshold of SUV max.

### Radiomics Features Analysis

A total of 45 radiomics parameters were extracted from images and divided into seven categories, including conventional PET/CT parameters (SUV and TLG), Histogram (HISTO), Shape value, Gray Level Co-occurrence Matrix (GLCM), Gray-Level Run Length Matrix (GLRLM), Neighborhood Gray-Level Different Matrix (NGLDM), Gray-Level Zone Length Matrix (GLZLM). The conventional PET/CT parameters included the minimum, average, maximum Standardized Uptake Value (SUV), and the Total Lesion Glycolysis (CONV_TLG) in the Volume of Interest. HISTO reflects the gray level of ROIs, regarding the “number of gray level” as “the size of bin”. Shape value is the sum total of volume of ROIs in mL and in voxels. GLCM describes the gray-level value distribution of voxel pairs along from 13 different directions at different distances in the ROI. GLRLM corresponds to the amount of homogeneous in 13 directions of the ROIs. NGLDM reflects the difference of gray-level between one voxel and its 26 neighbors in three dimensions. GLZLM describes the size of homogeneous zones for each gray-level in three dimensions.

The Mann-Whitney U test (U test) and χ2 test were used for comparing the baseline characteristics of those two groups. Since not all the parameters contributed to differentiating HCC and HL, we resorted to the results of operation characteristic curve (ROC curve) to select the most discriminative parameters in each category (Table 2). The binary logistic regression was used to transform the group of related parameters into a set of corresponding variables by three models. The discrimination ability of these models based on image-based parameters, texture parameters, and the combination of the two parameters were measured by the Area Under Curve (AUC) of ROC curves. *P*-values <0.05 were considered to be statistically significant. All statistical analyses were performed by SPSS (version 25, IBM, USA).

## Result

### Patients

The characteristics of patients and lesions were summarized in Table 1. There were 74 men (mean age, 54 years ± 14.5; age range 22–86 years) and 25 women (mean age, 52 years ± 16.3; age range 19–76 years). Based on histopathological proof, 23 patients had HL (10 PHL, 13 SHL) and 76 patients had HCC were prospectively included. The median ages of patients with HL was 51 and that of HCC was 54. Based on the TNM classification of malignant tumors, patients with HCC were divided into four groups, one patient of I stage, 16 of II stage, 14 of III stage, and 45 of IV stage. Among patients with HL, there were 12 Diffuse large B cell lymphoma (DLBCL), four B cell lymphoma (except DLBCL), six Hodgkin lymphoma, and one NK/T-cell lymphoma.

**Table 1:** Patient characteristics.

	**HL (*N* = 23) Median (Range or %)**	**HCC (*N* = 76) Median (Range or %)**	***P*-value**
**Gender**			
Male	10	64	<0.05
Female	13	12	
Age	51 (19–85)	54 (23–86)	0.878
**Histopathologic diagnosis of HL**			
Diffuse large B cell lymphoma (DLBCL)	12 (52%)	NA	NA
B cell lymphoma (except DLBCL)	4 (17%)		
Hodgkin lymphoma	6 (26%)		
NK/T-cell lymphoma	1 (5%)		
**TNM Stage**			**SUV mean (sd)**
I	NA	1	2.67
II		16	3.41 (0.96)
III		14	3.63 (1.33)
IV		45	4.82 (1.90)
all		76	4.27 (1.75)
**Ann Arbor Stage**			**SUV mean (sd)**
II	2	NA	4.79 (0.50)
IV	21		6.17 (5.05)
all	23		6.05 (4.83)

*HL, hepatic lymphoma; HCC, hepatocellular carcinoma; NK, natural killer; NA, not applicable; sd, standard deviation*.

**Table 2:** The results of ROC analysis of optimal image-based and texture parameters in PET and CT images for hepatocellular carcinoma vs. hepatic lymphoma.

	**HCC**		**HL**		**AUC**	***P*-value**
	**Median**	**Range**	**Median**	**Range**		
**Image-based parameters**						
CONVENTIONAL_SUVmin (SUV)	2.28	1.06–4.64	3.73	0.89–9.87	0.642	0.039
CONVENTIONAL_TLG (mL)	751.67	8.22–4403.85	552.96	11.50–6299.10	0.686	0.007
**Texture parameters**						
SHAPE_Compacity	2.53	0.77–5.78	1.52	0.00–6.13	0.784	<0.001
GLCM_Correlation	0.63	0.20–0.86	0.52	0.20–0.78	0.726	0.001
GLRLM_GLNU	238.79	8.58–2777.08	104.75	3.22–1622.28	0.774	<0.001
NGLDM_Contrast	0.08	0.01–0.46	0.22	0.03–1.42	0.721	0.001
GLZLM_GLNU	21.53	1.00–148.42	13.83	1.25–121.32	0.704	<0.001

*GLCM, gray-level co-occurrence matrix; GLRLM, gray-level run-length matrix; GLNU, Gray-Level Non-Uniformity; NGLDM, Neighborhood Gray-Level Different Matrix; GLZLM, gray-level zone-length matrix*.

### Imaging Features

**Figure 2:**
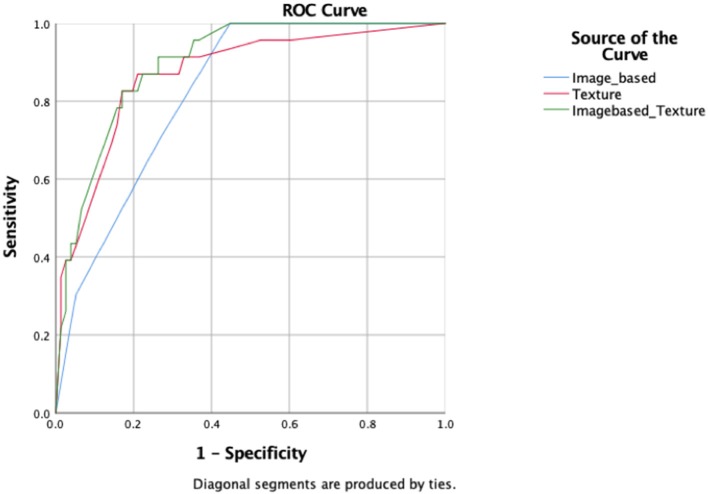
ROC curves of the three radiomic predictive models.

A total of six image-based parameters and 39 texture parameters were extracted and compared. The top two image-based parameters (CONVENTIONAL_SUVmin, CONVENTIONAL_TLG, AUC: 0.642, 0.686, *p* < 0.05) and the top five discriminative texture parameters (SHAPE_Compacity, GLCM_Correlation, GLRLM_GLNU, NGLDM_Contrast, and GLZLM_GLNU, AUC: 0.784, 0.726, 0.774, 0.721, 0.704, *p* < 0.05) were selected by ROC analysis (Supplementary Table 1). Binary logistic regression was used to transform groups of parameters into correspondent predictive models, including models transformed from image-based parameters, texture features, and the combination of those two kinds of parameters. Three predictive models were shown in Table 3: MODimage, MODtexture, and MODcombination. Table 4 showed the ROC results of these three models. AUC of the model transformed from image-based parameters was 0.822, with sensitivity of 69.6%, specificity of 73.7%. AUC of the model related with texture parameters was 0.870, with increased sensitivity of 91.3% and specificity of 77.6%. AUC of model transformed from the combination of image-based parameters and texture parameters was 0.898, with the same sensitivity and specificity as that of texture-based model (Figure 2).

**Table 3:** Regression models composed of image-based parameters, texture features, and the combination of those two kinds of parameters.

**Model**	**Formula**
MODimage	−2.154 CONVENTIONAL_SUVmin + 2.349 CONVENTIONAL_TLG - 1.065
MODtexture	20.405 SHAPE_Compacity-0.031 GLCM_Correlation+0.888 GLRLM_GLNU-2.498 NGLDM_Contrast-18.289 GLZLM_GLNU-0.758
MODcombination	36.534 SHAPE_Compacity+0.122 GLCM_Correlation+0.926 GLRLM_GLNU-1.783 NGLDM_Contrast-16.767 GLZLM_GLNU-0.975 CONVENTIONAL_SUVmin-17.756 CONVENTIONAL_TLG-0.76

**Table 4:** Comparison of differential diagnostic ability of the three predictive models.

**Test result variable(s)**	**Sensitivity**	**Specificity**	**AUC (95% CI)**	**Asymptotic Sig.b**
Image based	0.696	0.737	0.822(0.740–0.904)	<0.001
Texture	0.913	0.776	0.870(0.788–0.953)	<0.001
Combination	0.913	0.776	0.898(0.838–0.959)	<0.001

## Discussion

In this study, we used image-based parameters and texture parameters from 18F-FDG PET/CT to differentiate HCC from HL, and found many parameters significantly different between those two diseases. By comparing AUC of diagnostic models of image-based parameters, texture parameters, and the combination of the two parameters, we found that texture parameters presented better diagnostic ability than image-based parameters and that combination of the two parameters possessed a more effective diagnostic capacity than the other two groups.

The differentiation between HCC and HL is necessary, as their managements are different. Early stage HCC can be treated by excision of lesions, while the main treatment choice for secondary HL is multiagent chemotherapy (2). As an invasive method, the liver biopsy is prone to selection bias and may cause metastasis though it can offer reliable proofs for cancer diagnosis (13). The traditional radiological diagnosis could provide images with summarized features of the lesion. For instance, on contrast-enhanced images, HCC is characterized by “wash-out” pattern and fibrous tumor capsule (13). However, the images of HL are less specific and too variable to provide solid evidence for clinic diagnosis (4). Consequently, non-invasive and precise methods are required to differentiate HCC between HL.

PET/CT can reveal the metabolic characters of organs, and as tumor cells have enhanced glycolysis, they have higher 18F-FDG uptake compared with normal tissue. Previous studies have indicated that the SUV metrics of lymphoma was higher than that of carcinoma in PET/CT images when differentiating renal carcinoma and lymphoma with renal involvement (14, 15). However, a study claimed that the SUV max was not conclusive when distinguishing between primary nasopharyngeal lymphoma and nasopharyngeal carcinoma (16). Though many kinds of lymphoma are FDG avid, the diagnostic capacity of PET/CT remains unconfirmed (8). In our study, the SUV min of HL was higher than that of HCC while the TLG of HL was lower than that of HCC. The SUV min, a measurement of metabolic activity per body weight, could reflect the lowest point of metabolic activity within the tumor, but TLG takes into account the volume of the tumor lesion additionally. Therefore, this result may be affected by the volume of HCC.

Texture analysis can quantify image features by extracting the distribution and relation of pixel or voxel grayscale in images. Some studies have applied texture analysis to classifying benign and malignant liver lesions or stratifying different stages of liver fibrosis (17–19). However, no study has used texture analysis to distinguish HCC from HL, and it may because the morbidity of HL is relatively low, and the similarity of malignant lesions is more than that of benign and malignant liver lesions. In our study, texture parameters are more effective than image-based parameters in differentiation HCC from HL (AUC: 0.822 VS. 0.870). Previous studies compared carcinoma with lymphoma via texture analysis and proposed that the pixel gray-level value has a tight correlation with diagnosis, which is further confirmed by our results (20, 21). Early texture analysis in CT to differentiate malignant and benign liver lesion found that the First Order Statistics (FOS) performed best (22). However, in our study, FOS is less effective, while the secondary features, GLCM_Correlation, GLRLM_GLNU, NGLDM_Contrast, and GLZLM_GLNU, presented more significant differences between these two diseases. The result demonstrated that the gray levels of ROIs of HCC and HL were not distinguishing enough, and thus the second-order features such as gray-level value comparison were necessary.

Based on the better diagnostic capacity of texture parameters, we hypothesized that the combination of image-based and texture parameters contributes more to the clinic diagnosis of cancers. The result of AUC indicated that the combination group improved diagnostic capacity (AUC: 0.898), though the sensitivity of the specificity of the combination model remained the same as that of the texture model. Consistent with our previous studies, the same results were found in the diagnosis of breast carcinoma and breast involved lymphoma (23). Besides, another study found the same result in the differentiation of benign and malignant breast tumors (24). The combination of texture and image-based parameters could quantify and enhance the accuracy of the imaged-based PET/CT diagnosis.

However, the limited number of lymphoma group which did not include all kinds of lymphoma may lead to selected bias and therefore impact the accuracy of our result. Moreover, characterized as labor-intensive, ROIs are subject to manual measurement errors when compared with rectangular ROIs used drawn by computers. Finally, the relationship between texture parameters and histopathological structures requires further study.

In conclusion, our study confirmed the role of texture analysis in diagnosing different pathological cancer types and therefore proposed a new method for differentiating HCC and HL. Although both image-based and texture parameters can distinguish HCC from HL, the latter one is more efficient and the combination of the two parameters contribute to the diagnosis of HCC and HL more effectively.

## Data Availability

The datasets generated for this study are available on request to the corresponding author.

## Author Contributions

XM and HX contributed to the conception and design this study. WG and XC developed the methodology. XO and HZ analyzed and interpreted the data. YZ, YX, and TZ wrote the manuscript and approved the final submission of the study. All authors read and approved the final manuscript.

### Conflict of Interest Statement

The authors declare that the research was conducted in the absence of any commercial or financial relationships that could be construed as a potential conflict of interest.
